# It’s All About Timing: Early Feeding Promotes Intestinal Maturation by Shifting the Ratios of Specialized Epithelial Cells in Chicks

**DOI:** 10.3389/fphys.2020.596457

**Published:** 2020-12-17

**Authors:** Naama Reicher, Tal Melkman-Zehavi, Jonathan Dayan, Zehava Uni

**Affiliations:** Department of Animal Science, The Robert H. Smith, Faculty of Agriculture, Food and Environment, The Hebrew University of Jerusalem, Rehovot, Israel

**Keywords:** intestine, first feeding, epithelial cells, chick, maturation

## Abstract

The small intestine (SI) of chicks (*Gallus gallus*) matures rapidly during the initial post-hatch period and acquires digestive, absorptive, and secretive capabilities. The effects of the timing of first feeding on the quantities and distribution of specialized epithelial cells, which generate and maintain SI morphology and functionality, have not yet been examined. In this study, we identified specialized SI epithelial cell sub-types, including stem, progenitor, proliferating, and differentiated cells within crypts and villi of chicks during the first 10 days post-hatch, by *in situ* hybridization (ISH), immunofluorescence (IF), and histochemical staining. We then examined their quantities and ratios between day of hatch and d10 in chicks that were fed upon hatch [early feeding (EF)], compared to chicks that were fed 24 h post-hatch [delayed feeding (DF)]. Results showed that EF increased total cell quantities in the crypts and villi at days 1, 3, 7, and 10, compared to DF (*p* < 0.0001). At d3, EF, in comparison to DF, decreased crypt stem cell proportions (*p* < 0.0001), increased crypt proliferating (*p* < 0.01) and differentiated (*p* < 0.05) cell proportions, and increased villus enterocyte proportions (*p* < 0.01). By d10, EF increased both the quantities and proportions of villus enterocytes and goblet cells, compared to DF. We conclude that feeding upon hatch, compared to 24 h-delayed feeding, enhanced SI maturation and functionality by increasing the quantities and proportions of proliferating and differentiated cells, thus expanding the digestive, absorptive, and secretive cell populations throughout the initial post-hatch period.

## Introduction

The small intestinal (SI) mucosa is lined with a specialized epithelium, comprised of various cell sub-types, which undergo constant renewal and change. Functional SI epithelial cells are organized in lumen-facing villi, and are responsible for nutrient digestion and absorption, mucin secretion, chemosensation, and hormonal signaling. Intestinal stem cells reside in crypts at the bottom of each villus, and maintain a balance between production of progenitor cells and self-renewal. Progenitor cells proliferate, differentiate, and migrate along the crypt-villus axis, giving rise to all functional cells (Potten and Loeffler, 1990; Gehart and Clevers, 2019). Cellular lineages, niche characterization and regulatory processes within the SI epithelium have been widely researched in human and mouse models (Blache et al., 2004; Barker et al., 2007; Carulli et al., 2014; Moor et al., 2018; Bahar Halpern et al., 2020). However, in chicken, cell sub-type dynamics within the SI epithelium is generally under-characterized. Few studies have indicated both similarities and differences in the spatial organization of the SI epithelial cell sub-types between chicken and humans or mice. For example, in a manner similar to mice and humans, stem cells are restricted to the crypts and enterocytes are found along the villi in chicken (Zhang and Wong, 2017, 2018). However, in contrast to mice and humans, proliferating and immune cells in chicken were observed along the villi, as well as within the crypts (Uni et al., 1998; Wang et al., 2016; Bar Shira and Friedman, 2018). Being a precocial species, newly hatched chicks undergo intensive intestinal maturation processes, in order to make a rapid transition from egg yolk-based nutrition to external feeding (Noy and Sklan, 1998; Uni et al., 1999; Yadgary et al., 2010). This transition occurs during the initial 10 days post-hatch and is governed by morphological, cellular, and molecular changes in the SI epithelium, including cell proliferation, formation of crypts, and elongation of villi. Functional cells develop along the villi, and mainly consist of enterocytes, expressing nutrient transporters and digestive enzymes, and protective mucin-secreting goblet cells, in a 5:1 ratio (Uni et al., 2000, 2003a,b; Gilbert et al., 2007; Zwarycz and Wong, 2010; Speier et al., 2012). SI development and functionality during this initial 10 days period is highly correlated with long-term performance parameters, including feed intake, body weight, and mortality rates (Sklan, 2001). In poultry production, chicks are susceptible to 24–72 h delays in the timing of first feeding, due to broad hatching windows, hatchery treatments, and transportation. Research shows that delayed first feeding in chicks disrupts SI development by limiting crypt depths and villus heights, altering epithelial proliferation patterns, dysregulating differentiation-associated genes, and disrupting mucin dynamics (Geyra et al., 2001, 2002; Uni et al., 2003a, de Jong et al., 2017). Long-term systematic effects of delayed first feeding include impaired nutrient utilization, altered regulation of metabolic pathways, disrupted barrier functions, and impaired gut immunity, all of which jeopardize animal welfare, impair performance in increase mortality rates (Bigot et al., 2003; Bar Shira et al., 2005; de Jong et al., 2017; Hicks et al., 2019; Payne et al., 2019; Proszkowiec-weglarz et al., 2020). In this study, we aimed to evaluate how the timing of first feeding of chicks influences the quantities and distribution of specialized SI epithelial cell sub-types during the initial 10 days post-hatch. Cell sub-types examined were stem, progenitor, proliferating, and differentiated cells, which provide basic input for the capacity of the SI to generate and maintain digestive, absorptive, and immune functionality. We located these cell sub-types using *in situ* hybridization (ISH) for the stem cell marker leucine-rich repeat-containing G-protein coupled receptor 5 (Lgr5; Barker et al., 2007) and enterocyte marker Peptide transporter 1 (PepT1; Fei et al., 2000); immunofluorescence (IF) for the stem/progenitor cell marker SRY-box transcription factor 9 (Sox9; Blache et al., 2004), the proliferation marker proliferating cell nuclear antigen (PCNA; Kubben et al., 1994) and enterocyte marker fatty acid binding protein (FABP; Storch and Corsico, 2008); and histochemical staining for goblet cells. We then examined the effects of immediate post-hatch access to feed [early feeding (EF)] and a 24 h delay in the timing of first feeding [delayed feeding (DF)], corresponding to the minimal delay of first feeding in current poultry commercial practice, on specific cell sub-type abundances and ratios throughout the critical first 10 days post-hatch period.

## Materials and Methods

### Animals and Experimental Design

Fertile Cobb500 broiler eggs (*n* = 70) were obtained from a commercial hatchery (Brown Ltd., Hod-Hasharon, Israel) at day of lay and incubated in a Petersime hatchery at the Faculty of Agriculture of the Hebrew University, under standard conditions (37.8°C, 60% relative humidity) for 21 days. Hatching window was monitored from the beginning of embryonic day 20 (e20). Fifty four chicks of equal weights (42.2 ± 3.2 gr SEM), that hatched between e20.5 and e21, were selected for the experimental procedures. Unhatched eggs (12% of total incubated eggs) and chicks which hatched after the end of e21 were excluded from the experiment. At hatch, six chicks were processed for histological procedures. The remaining 48 chicks were transferred to brooders at the Faculty of Agriculture of the Hebrew University and were randomly divided into two groups, each subject to a different timing of first feeding: EF chicks received initial access to feed and water immediately upon arrival to brooder (day of Hatch) and DF chicks received initial access to feed and water 24 h after arrival to brooder (d1). Both groups were fed with a standard commercial starter diet (Brown feedmill, Kaniel, Israel), formulated according to NRC (National Research Council, 1994) recommendations. After granting initial access to feed, both groups were fed ad-libitum. Tissue sampling for histological procedures was conducted at days 1, 3, 7, and 10 on six chicks from each group.

### Tissue Sampling

Sampled chicks were euthanized by CO_2_, according to established guidelines for animal care and handling and were approved by the Hebrew University Institutional Animal Care and Use Committee (IACUC:AG-17-15355-2). The SI jejunum segment (1 cm piece from the midpoint between the duodenal loop and Meckel’s diverticulum) was immediately excised from each chick, rinsed in phosphate buffered saline (PBS), and fixed in 3.7% formaldehyde in PBS (pH 7.4) for 24 h at room temperature (RT). Tissues was then rinsed out in PBS, dehydrated in grated series of ethanol, cleared by Histochoice® (Sigma-Aldrich, Rehovot, Israel) and embedded in Paraplast® (Sigma-Aldrich, Rehovot, Israel). Tissue blocks were sectioned 5 μm thick with a microtome, and mounted on SuperFrost Plus™ glass slides (Bar-Naor Ltd., Petah-Tikva, Israel).

### *In situ* Hybridization

Jejunum sections were deparaffinized by Histochoice® (Sigma-Aldrich, Rehovot, Israel) and rehydrated in a graded series of ethanol. RNAscope® ISH was performed as described by Wang et al. (2012), using custom made probes and commercial kits (ACD, Newark, CA) according to the manufacturer’s protocol. Lgr5 mRNA transcripts were hybridized using a Gg-Lgr5 probe (XM_425441.4, Cat. No. 480781) and detected using RNAscope 2.5 HD Kit-RED (Cat. No. 322350). PepT1 mRNA transcripts were hybridized using a Gg-SLC15A1 probe (NM_204365.1, Cat. No. 462341) and detected using RNAscope 2.5 HD Kit-BROWN (Cat. No. 322300). All slides were counterstained with 50% hematoxylin (Sigma-Aldrich, Rehovot, Israel). Results were validated using a positive control probe (Gg-PPIB, Cat. No 453371) and a negative control probe DapB (Cat. No. 310043).

### Histochemical and Immunofluorescent Staining

Jejunum sections were deparaffinized by Histochoice® (Sigma-Aldrich, Rehovot, Israel) and rehydrated in a graded series of ethanol. For goblet cell quantification, slides were stained with Alcian blue (AB; A5268, Sigma-Aldrich, Rehovot, Israel) and periodic acid Schiff (PAS; PAS staining kit 395B, Sigma-Aldrich, Rehovot, Israel), and sealed with Fluoromount G with 4′,6-diamidino-2-phenylindole (DAPI; Invitrogen, 00-4959). For immunostaining, antigen retrieval was performed by heating slides for 20 min in pH5.5 citrate buffer (Sigma-Aldrich, Rehovot, Israel). Sections were permeabilized in PBS with 0.1% Tween® (Sigma-Aldrich, Rehovot, Israel; PBST), and blocked in 1% bovine serum albumin (BSA, Sigma-Aldrich, Rehovot, Israel) in PBS. Slides were incubated overnight at 4°C with primary antibodies in two separate combinations: (1) Rabbit anti-Sox9 (1:150, AB5535, Millipore) and mouse anti-PCNA (1:300, sc-56, Santa Cruz); (2) Goat anti-FABP (1:250, ab60272, Abcam) and mouse anti-PCNA (1:300, sc-56, Santa Cruz). Following washes in PBS and PBST, slides were incubated 1 h at RT with the following secondary antibodies: (1) Sox9: Donkey anti-Rabbit Alexa Fluor 488 (1:100, 711-545-152, Jackson ImmunoResearch Laboratories, Inc); PCNA: Donkey anti-mouse Cy3 (1:100, 715-165-150, Jackson ImmunoResearch Laboratories, Inc) and (2) FABP: Donkey anti-Goat Alexa Fluor 488 (A-11055, Invitrogen); PCNA: same as in (1). Slides were then washed in PBS and sealed with Fluoromount G with DAPI (00-4959, Invitrogen).

### Image Acquisition and Processing

Images were acquired using a BX40 Olympus microscope (Waltham, MA, United States) wide-field microscope, connected to a DP73 camera, and processed in Cell Sense Imaging Software (version 1.16), using X10 and X40 objectives. Whole villi images were acquired by EVOS® FL Auto Imaging System (Thermo Fisher Scientific) using a X20 objective and image stitching. Images were post-processed for brightness/contrast adjustments using ImageJ software. RNAscope® ISH probe density per μm^2^ was calculated using HALO® software (version 2.2.1870). Villus and crypt cells were quantified by counting DAPI-stained nuclei in at least 10 villi and 10 crypt cells per chick (total of at least 30 villi and 30 crypt cells per group at each timepoint). Villus/crypt cell ratios were calculated by dividing the number of cells per villus by the number of cells per adjacent crypt in 6–7 villus-crypt units per chick (total of at least 20 units per group at each timepoint). Immunofluorescent cells within each villus (either FABP^+^/PCNA^+^ or FABP^+^/PCNA^−^) and within each crypt (either Sox9^+^/PCNA^−^, Sox9^+^/PCNA^+^, Sox9^−^/PCNA^+^, or Sox9^−^/PCNA^−^) were quantified in at least 10 villi and 10 crypt cells per chick (total of at least 30 villi and 30 crypt cells per group at each timepoint). Cell sub-type ratios were calculated by dividing the number of each cell sub-type by the number of DAPI-stained villus/crypt cells. Goblet cells were quantified by AB-PAS staining in at least 10 villi and 10 crypt cells per chick (total of at least 30 villi and 30 crypt cells per group at each timepoint), and their ratios were calculated by dividing their quantities by the number of DAPI-stained villus/crypt cells. Image acquisitions and processing were conducted by two unblinded observers.

### Statistical Analysis

Data were analyzed using one-way ANOVA. Differences were considered significant at *p* < 0.05. Student’s *t*-test was conducted for detecting significant differences between EF and DF groups at each timepoint and significant differences were marked with an asterisk. Tukey-Kramer HSD test was conducted for detecting significant differences between ages within each group and significant differences were marked with different lowercase letters. Graphical data were expressed as mean ± SEM. All statistical analyses were conducted with JMP Pro 14 software (SAS Institute, Cary, NC, United States).

## Results

**Figure 1:**
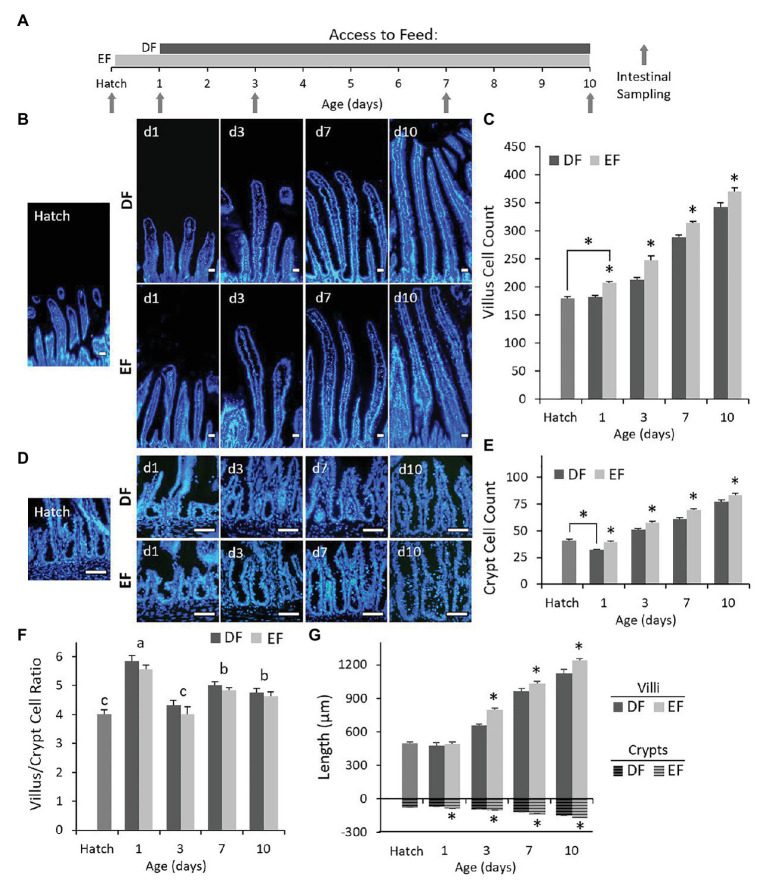
Early feeding increases crypt and villi cell counts throughout post-hatch intestinal development. **(A)** Experimental timeline. Chicks at hatch were divided into two groups: EF chicks were fed upon hatch and delayed feeding chicks were fed 24 h post-hatch. Intestinal segments from both groups were sampled at Hatch (prior to feeding in both groups), d1 (prior to feeding in DF chicks), d3, d7, and d10. Villus **(B)** and crypt **(D)** cells were stained with 4',6-diamidino-2-phenylindole (DAPI) from Hatch to d10 in DF and EF chicks. Scale bars are 50 μm. DAPI-stained villus **(C)** and crypt **(E)** cells were counted from Hatch to d10 in DF and EF chicks. Values are means + SEM, asterisks mark significant differences between DF and EF chicks for each age by *t*-test, *p* < 0.05. **(F)** Ratios between villi cell counts and crypt cell counts from Hatch to d10 in DF and EF chicks. Ratios did not differ between DF and EF chicks. Letters mark significant differences between ages in values from both groups by Tukey-Kramer test, *p* < 0.05. **(G)** Measurements of villi heights (solid-filled bars) and crypt depths (striped-bars) from Hatch to d10 in DF and EF chicks. Values are means + SEM, and asterisks mark significant differences between DF and EF chicks for each age by *t*-test.

Our experimental procedures were conducted on SI jejunum sections from chicks between the age of Hatch and day 10 (d10) belonging to two groups: EF chicks began feeding upon hatching and DF chicks began feeding 24 h post-hatch (Figure 1A). In order to evaluate the effects of the timing of first feeding on SI post-hatch development through morphological parameters, we quantified the total number of cells by DAPI nuclear staining within villi (Figure 1B) and crypts (Figure 1D) and measured villi heights and crypt depths (Figure 1G) in both groups between Hatch and d10. At d1, villus and crypt cell counts were significantly higher in EF chicks, compared to DF chicks (*p* < 0.0001 for both; Figures 1C,E). Interestingly, the total number of crypt cells in EF chicks at d1 was similar to that of chicks at Hatch, while DF chicks at d1 exhibited a significant decrease in crypt cell counts in comparison to chicks at Hatch (*p* < 0.0001; Figures 1C,E). Crypt depth measurements were decreased accordingly in DF chicks compared to EF chicks (*p* < 0.0001) and chicks at Hatch (*p* = 0.0147; Figure 1G). In contrast, the number of cells per villus at d1 in comparison to Hatch was significantly higher in EF chicks, but did change in DF chicks (*p* = 0.0043; Figure 1C). Surprisingly, villi lengths did not differ among DF chicks, EF chicks, and chicks at Hatch (Figure 1G). From d3 to d10, we observed an increase in villus and crypt cell counts in both groups, with cell numbers being consistently higher in EF chicks, compared to DF chicks, at all timepoints (villi: d1, d3, and d7, *p* < 0.0001; d10, *p* = 0.0027; crypts: d1, *p* < 0.0001; d3, *p* = 0.003; d10, *p* = 0.0067; Figures 1C,E). Villi lengths and crypt depths increased accordingly (crypts: d3, *p* = 0.003; d7, *p* < 0.0001; d10, *p* = 0.0067; villi: d3, *p* < 0.0001; d7, *p* < 0.0235, d10, *p* = 0.0032; Figure 1G). The villus height to crypt depth ratio is an indicator of SI development, which may be measured through several methods (Wilson et al., 2018). Since this ratio represents the replenishment and turnover rate of differentiated villus cells (Potten and Loeffler, 1990), we calculated the villus cell to crypt cell ratio in order to assess the effects of the timing of first feeding on villus cell accumulation relative to crypt development. Results showed that at all timepoints, timing of first feeding did not affect the villus/crypt cell ratio (Figure 1F). However, a comparison of villus/crypt cell ratios from both groups between ages revealed significant changes with age: villus/crypt cell ratios were lowest at Hatch and d3, highest at d1 and reached a stable, intermediate level at d7, which was maintained at d10 (*p* < 0.0001; Figure 1F).

**Figure 2:**
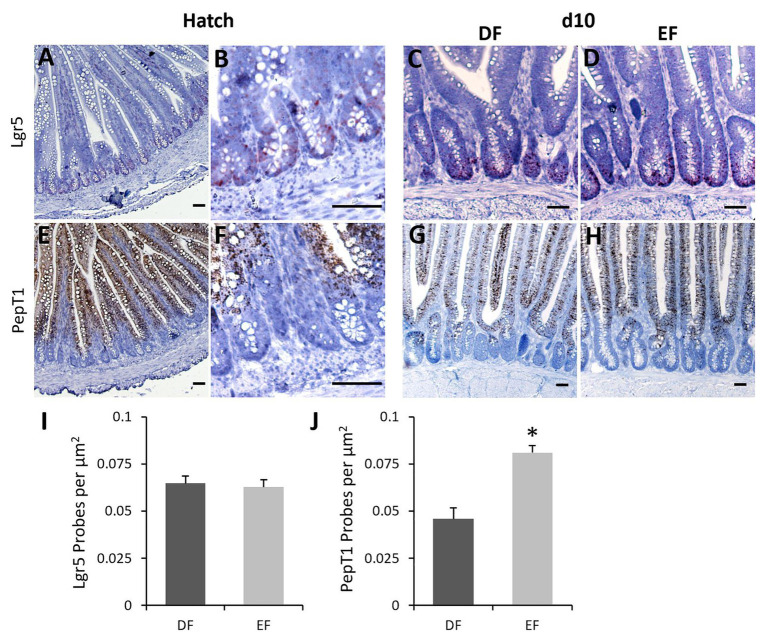
Early feeding increases villus PepT1 expression and does not affect crypt Lgr5 expression at d10. mRNA expression of the stem cell marker Leucin-rich repeat-containing G-protein coupled receptor 5 (Lgr5) detected by RNAscope *in situ* hybridization (ISH) at Hatch (**A**, X100 magnification; **B**, crypt zone by X400 magnification) and d10, in DF **(C)** and EF **(D)** chicks (X400 magnification) is restricted to crypts at both timepoints. Probes were detected with fast-red, and tissues were counterstained with 50% hematoxylin. mRNA expression of the enterocyte marker Peptide transporter 1 (PepT1) detected by RNAscope (ISH) at Hatch (**E**, X100 magnification; **F**, crypt zone by X400 magnification) and d10, in DF **(G)** and EF **(H)** chicks (X100 magnification) is restricted to villi at both timepoints. Probes were stained with 3,3'-diaminobenzidine (DAB), and tissues were counterstained with 50% hematoxylin. Scale bars indicate 50 μm. At d10, Lgr5 probe densities in DF and EF chicks were calculated relative to crypt area **(I)**, and PepT1 probe densities in DF and EF chicks were calculated relative to villus area **(J)**. Values are means + SEM, and asterisks mark significant differences by *t*-test, *p* < 0.05.

**Figure 3:**
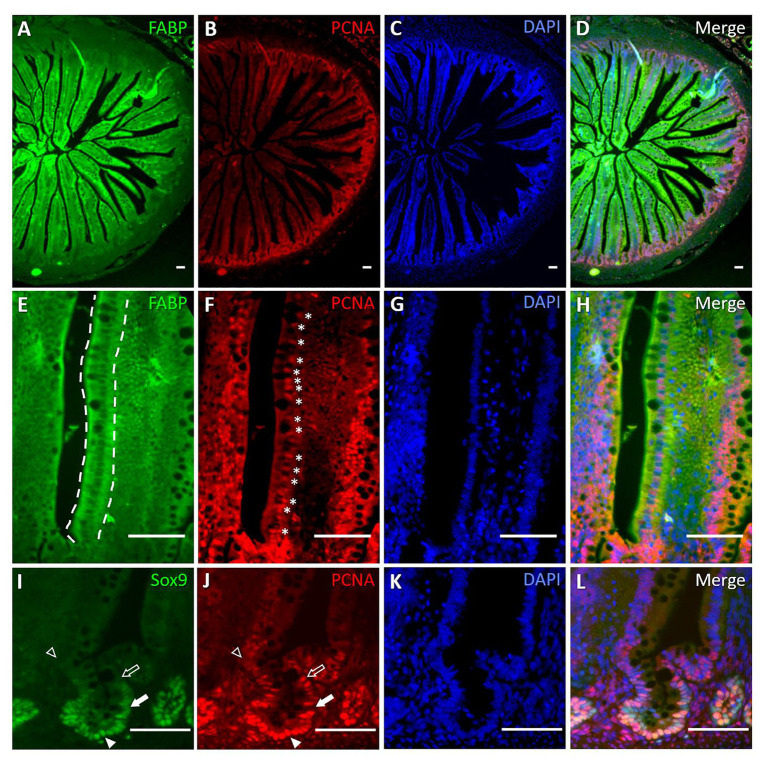
Stem, progenitor, proliferating, and differentiated cells exhibit specific localization within villi and crypts at Hatch. Immunofluorescence (IF) of enterocyte marker fatty acid binding protein (FABP), proliferating cell nuclear antigen (PCNA), DAPI nuclear staining, and their merge at X100 magnification **(A–D)**, and X400 magnification **(E–H)**, and stem/progenitor cell marker SRY-Box Transcription Factor 9 (Sox9), PCNA, DAPI, and their merge **(I–L)** at Hatch. FABP expression was localized to all villus cells (**E**, dashed outlines). Proliferating cells were found along the villus (**F**, asterisks), overlapping FABP^+^ cells. Sox9 expression was restricted to crypts **(I)**. Arrows indicate Sox9^+^/PCNA^−^ cells, arrowheads indicate Sox9^+^/PCNA^+^ cells, arrow outlines indicate Sox9^−^/PCNA^+^ cells, and arrowhead outlines indicate Sox9^−^/PCNA^−^ cells **(I,J)**.

**Figure 4:**
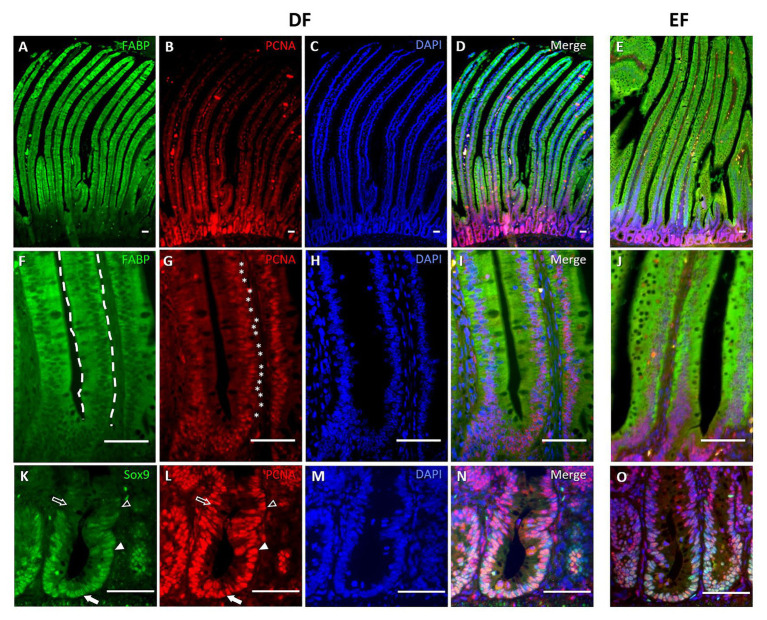
Stem, progenitor, proliferating, and differentiated cells exhibit specific localization within villi and crypts at d10. IF of enterocyte marker FABP, PCNA, DAPI nuclear staining, and their merge at X100 magnification **(A–D)**, and X400 magnification **(F–I)**, and stem/progenitor cell marker Sox9, PCNA, DAPI, and their merge **(K–N)** in DF chicks at d10. Merged immunofluorescent images of the same markers in EF chicks at d10 are presented in the right, separate panel: FABP and PCNA at a magnification of X100 **(E)** and X400 **(J)**; Sox9 and PCNA at X400 magnification **(O)**. FABP expression was localized to all villus cells (**F**, dashed outlines). Proliferating cells were found along the villus (**G**, asterisks), colocalizing with FABP^+^ cells. Sox9 expression was restricted to crypts **(K)**. Arrows indicate Sox9^+^/PCNA^−^ cells, arrowheads indicate Sox9^+^/PCNA^+^ cells, arrow outlines indicate Sox9^−^/PCNA^+^ cells, and arrowhead outlines indicate Sox9^−^/PCNA^−^ cells **(K,L)**.

Next, we examined expression patterns of stem cell marker Lgr5 (Barker et al., 2007) and enterocyte marker PepT1 (Fei et al., 2000) by RNAscope® ISH, which allows specific and sensitive quantification of mRNA expression in a spatial context (Wang et al., 2012). Consistent with previous findings (Zhang and Wong, 2017, 2018), Lgr5 mRNA expression was restricted to crypts (Figures 2A,B) and PepT1 mRNA expression was located along the villi (Figures 2E,F) at Hatch. At d10, Lgr5 expression was mainly localized to the bottom third of the crypts in both DF and EF chicks (Figures 2C,D), and PepT1 expression was similar to that at Hatch Probe (Figures 2G,H). Probe density quantification at d10 revealed that Lgr5 expression did not differ between DF and EF chicks (Figure 2I), yet PepT1 expression was significantly higher in EF chicks, compared to DF chicks (*p* < 0.0001; Figure 2J). These data suggest that by d10, EF enhances the enterocyte population within the villi, while the crypt stem cell population remains stable. Therefore, in order to examine whether the timing of first feeding affected cellular proliferation and differentiation from crypt-based stem cells into differentiated villi cells during post-hatch intestinal development, we examined co-localizations of stem/progenitor cell marker Sox9 (Blache et al., 2004), cytosolic enterocyte marker FABP (Storch and Corsico, 2008), and proliferation marker PCNA (Kubben et al., 1994) in crypt and villi cells by IF. We found that Sox9 expression was restricted to the bottom two-thirds of crypt cells, FABP was expressed exclusively in villi cells, and PCNA was co-expressed in cells of both compartments between Hatch and d10. Representative images from Hatch (Figure 3) and d10 DF chicks (Figure 4) demonstrate that at both timepoints, villi cells were all FABP^+^ (Figures 3A,E, 4A,F, dashed outlines), while some were also positive for PCNA (Figures 3B,F, 4B,G, asterisks), and crypt cells (Figures 3I,J, 4K,L) were either Sox9^+^/PCNA^−^ (arrows), Sox9^+^/PCNA^+^ (arrowheads), Sox9^−^/PCNA^+^ (arrow outlines), or Sox9^−^/PCNA^−^ (arrowhead outlines). Representative images from d10 EF chicks show a similar pattern of crypt and villi cell sub-type localization (Figures 4E,J,O).

**Figure 5:**
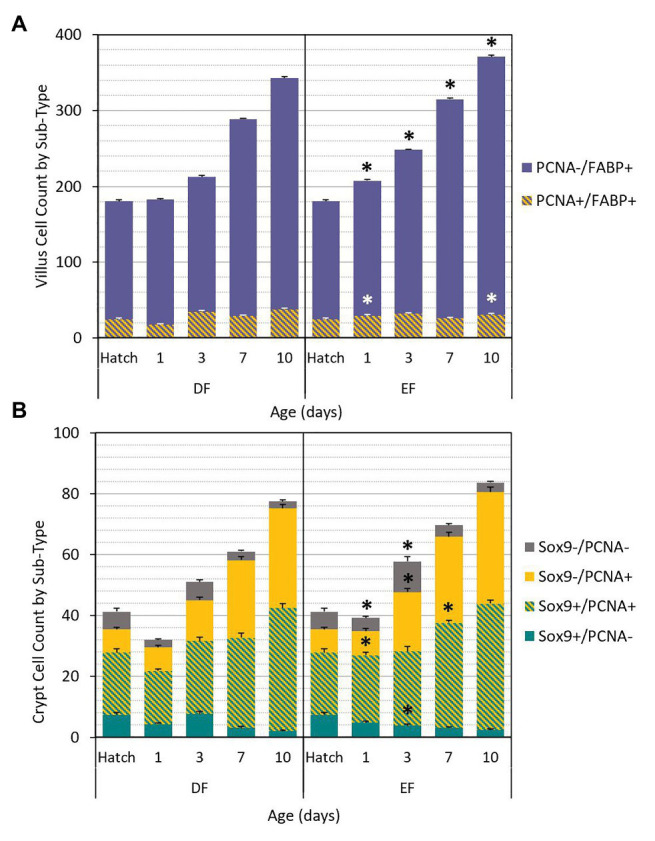
Villus and crypt cell sub-type counts are affected by the timing of first feeding. **(A)** Villus cell sub-type counts by IF between Hatch and d10 in DF chicks (left) and EF chicks (right). Cells were either FABP^+^/PCNA^–^ (purple bars), or FABP^+^/PCNA^+^ (lined bars). **(B)** Crypt cell sub-type counts by IF between Hatch and d10 in DF chicks (left) and EF chicks (right). Cells were either Sox9^+^/PCNA^−^ (teal bars), Sox9^+^/PCNA^+^ (lined bars), Sox9^−^/PCNA^+^ (yellow bars), or Sox9^−^/PCNA^−^ (gray bars). Values are means + SEM, asterisks mark significant differences between DF and EF at each age by *t*-test, *p* < 0.05.

**Figure 6:**
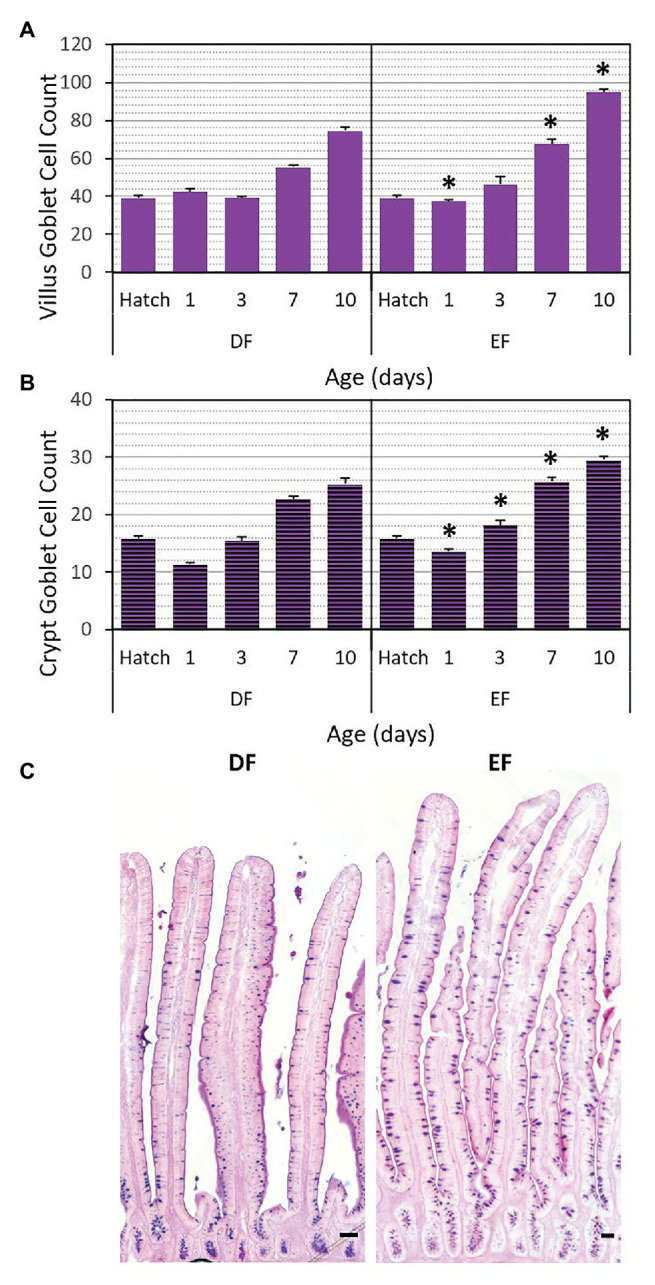
Villus and crypt goblet cell counts between Hatch and d10 are affected by the timing of first feeding. Goblet cells were quantified by counting Alcian blue-periodic acid Schiff (AB-PAS) mucin staining in villi **(A)** and crypts **(B)** between Hatch and d10 in DF and EF chicks. Values are means + SEM, and asterisks mark significant differences between DF and EF chicks for each age by *t*-test, *p* < 0.05. **(C)** Representative images of AB-PAS staining at d10 in DF and EF chicks. Scale bars indicate 50 μm.

Each of the two villus cell sub-types was quantified from the first FABP^+^ villus cell, located within the crypt-villus border, up to the villus tip (Figure 5A). Results showed that in EF chicks, compared to DF chicks, FABP^+^/PCNA^−^ cell counts were significantly higher at d1, d3, d7, and d10 (*p* < 0.0001, *p* < 0.0001, *p* = 0.0027; and *p* = 0.0011, respectively), while FABP^+^/PCNA^+^ cell counts were significantly higher at d1 (*p* < 0.0001) and significantly lower at d10 (*p* = 0.0011; Figure 5A). The ratios between villus cell sub-types (Table 1) were significantly affected by the timing of first feeding at all timepoints: at d1, FABP^+^/PCNA^−^ cell ratios decreased and FABP^+^/PCNA^+^ increased significantly (*p* = 0.0011), whereas at d3, d7 and d10, FABP^+^/PCNA^−^ cell ratios decreased and FABP^+^/PCNA^+^ increased significantly (*p* < 0.0001, *p* = 0.0027; and *p* = 0.0011, respectively) in EF chicks, compared to DF chicks (Table 1). In the crypts, the four cell sub-types were quantified from crypt base to crypt-villus border (Figure 5B). EF chicks, compared to DF chicks, exhibited significantly increased Sox9^+^/PCNA^+^ and Sox9^−^/PCNA^−^ cell counts at d1 (*p* < 0.0001 and *p* = 0.0012, respectively), significantly decreased Sox9^+^/PCNA^−^ and increased Sox9^−^/PCNA^+^ and Sox9^−^/PCNA^−^ cell counts at d3 (*p* = 0.0001, *p* = 0.0007, *p* = 0.044, respectively) and significantly increased Sox9^+^/PCNA^+^ cell counts at d7 (*p* = 0.0084). The ratio of each crypt cell sub-type was calculated as the fraction from total number of crypt cells (Table 1), and was found to be significantly affected by the timing of first feeding at d1, in which EF chicks exhibited significantly higher ratios of Sox9^−^/PCNA^−^ cells, compared to DF chicks (*p* = 0.015), and at d3, in which EF chicks exhibited significantly lower ratios of Sox9^+^/PCNA^−^ cells and significantly higher ratios of Sox9^−^/PCNA^+^ and Sox9^−^/PCNA^−^ cells, compared to DF chicks (*p* < 0.0001, *p* = 0.0086 and *p* = 0.0365, respectively; Table 1). Lastly, goblet cells were quantified within crypts and villi at all timepoints by counting cells stained with Alcian blue and periodic acid Schiff (AB-PAS), which identifies their intracellular and secreted acidic and neutral mucins (Forstner et al., 1994; Uni et al., 2003a; Figure 6). Villi goblet cell counts were significantly lower at d1 (*p* = 0.013) and significantly higher at d7 and d10 (*p* < 0.0001) in EF chicks, compared to DF chicks (Figure 6A). Their ratios, relative to the total number of villus cells, were accordingly lower at d1 (*p* < 0.0001) and higher at d7 (*p* = 0.0086) and d10 (*p* < 0.0001) in EF chicks, compared to DF chicks (Table 2). Crypt goblet cell counts in EF chicks, compared to DF chicks, were also significantly lower at d1 (*p* < 0.0001), yet were significantly higher at d3, d7 and d10 (*p* = 0.0159, *p* = 0.0068 and *p* = 0.0067, respectively; Figure 6B). However, their ratios, relative to the total number of crypt cells, were affected by the timing of first feeding at d3 and d10 only, with significant increases in EF chicks, compared to DF chicks (*p* = 0.0011 and *p* = 0.0013, respectively; Table 2).

**Table 1:** Villus and crypt cell sub-type ratios between Hatch and d10 in delayed feeding (DF) and early feeding (EF) chicks.

Age (days)	EF/DF	Villus cell ratios (%)		Crypts cell ratios (%)			
		FABP^+^/PCNA^+^	FABP^+^/PCNA^−^	Sox9^+^/PCNA^−^	Sox9^+^/PCNA^+^	Sox9^−^/PCNA^+^	Sox9^−^/PCNA^−^
Hatch	-	13.4 ± 1.1	86.6 ± 1.1	17.8 ± 2	49.7 ± 2.6	19 ± 1.8	13.5 ± 2.4
D1	DF	9.3 ± 0.8	90.7 ± 0.8	13.1 ± 1.3	55.6 ± 2	23.8 ± 1.7	7.5 ± 0.7
	EF	13.9 ± 1^*^	86.1 ± 1^*^	12.3 ± 1.4	57.3 ± 2.1	19.2 ± 1.8	11.2 ± 1.3^*^
D3	DF	16.2 ± 0.9	83.8 ± 0.9	15 ± 1.7	46.9 ± 2	26.1 ± 1.7	12 ± 1.3
	EF	12.9 ± 0.4^*^	87.1 ± 0.4^*^	6.5 ± 1.9^*^	42.6 ± 2.7	33.2 ± 1.9^*^	17.7 ± 2.9^*^
D7	DF	10.1 ± 0.4	89.9 ± 0.4	4.7 ± 0.8	48.3 ± 2.2	42.1 ± 1.9	4.9 ± 0.9
	EF	8.1 ± 0.5^*^	91.9 ± 0.5^*^	4.4 ± 0.4	49.9 ± 1.5	40.4 ± 1.7	5.5 ± 0.7
D10	DF	11 ± 0.5	89 ± 0.5	2.5 ± 0.5	52.1 ± 1.5	42.4 ± 1.3	3 ± 0.6
	EF	8.2 ± 0.6^*^	91.8 ± 0.6^*^	2.8 ± 0.4	49.9 ± 1.4	43.5 ± 1.4	3.8 ± 0.6

Ratios of cell sub-types, relative to the total number villus/crypt cells. Values are means ± SEM, asterisks, and bold lettering mark significant differences between DF chicks and EF chicks at each age by *t*-test, *p* < 0.05.

**Table 2:** Villus and goblet cell ratios between Hatch and d10 in DF and EF chicks.

Age (days)	EF/DF	Villus goblet cell ratio (%)	Crypts goblet cell ratio (%)
Hatch	-	21.5 ± 0.7	41.7 ± 1.2
D1	DF	23.4 ± 0.9	31.9 ± 1
	EF	18.1 ± 0.4^*^	33 ± 1
D3	DF	18.6 ± 0.4	30.9 ± 1.3
	EF	18 ± 1.2	37.4 ± 1.3^*^
D7	DF	19.2 ± 0.4	42.5 ± 1.2
	EF	21.5 ± 0.8^*^	40 ± 1.1
D10	DF	21.8 ± 0.6	41.7 ± 1.4
	EF	25.5 ± 0.4^*^	46.7 ± 0.9^*^

Ratios of villus and crypt goblet cells, relative to the total number villus/crypt cells. Values are means ± SEM, asterisks, and bold lettering mark significant differences between DF chicks and EF chicks at each age by *t*-test, *p* < 0.05.

## Discussion

In this study, we have shown that early first feeding of chicks promoted early maturation of the small intestinal (SI) epithelium by: (1) increasing the quantities and proportions of progenitor and differentiated crypt cells and (2) increasing the quantities and proportions of mature enterocytes in the villi. Our results show that the timing of first feeding of chicks alters SI morphology and functionality during the initial 10 days post-hatch through changes in the distribution of stem (Sox9^+^/PCNA^−^), progenitor (Sox9^+^/PCNA^+^), proliferating (Sox9^−^/PCNA^+^) and differentiated (Sox9^−^/PCNA^−^) cells in the crypts, and proliferating (PCNA^+^/FABP^+^), and differentiated cells (PCNA^−^/FABP^+^ and goblet cells) in the villi.

During the initial 10 days post-hatch, we identified Lgr5^+^ and Sox9^+^ stem cells that were restricted to crypts, and PepT1^+^ and FABP^+^ enterocytes that populated the villi. This distribution pattern is similar to that of the mouse and human SI epithelium (Barker et al., 2007; Gehart and Clevers, 2019). Sox9 is a Wnt target transcription factor, which is expressed in high levels by non-dividing stem cells, and is associated in low levels with progenitor cells (Bastide et al., 2007; Gracz et al., 2010; Bankaitis et al., 2018). In light of this, we divided crypt cells into four sub-types and characterized Sox9^+^/PCNA^−^ cells as non-dividing crypt-base stem cells, Sox9^+^/PCNA^+^ cells as progenitor cells, Sox9^−^/PCNA^+^ cells as proliferating cells and Sox9^−^/PCNA^−^ cells as differentiated, non-enteroendocrine cells.

Unlike humans and mice, in which proliferating cells are restricted to the crypt zone, proliferation marker PCNA in chicks was expressed both in crypt cells and along villi, within cells co-expressing FABP. The presence of proliferating cells within the SI villus has been previously documented in chicken of varying ages (Uni et al., 1998; Zhang et al., 2014), as well as in numerous other species, ranging from mammals (Verdile et al., 2019) to reptiles (Helmstetter et al., 2009; Borges et al., 2018), amphibians (Ikuzawa et al., 2006), and fish (Sklan et al., 2004; LØkka et al., 2013; Verdile et al., 2020). Species-dependent changes in feeding behavior and digestive physiology have been found to alter the quantities of villus proliferating cells, thus reflecting the regenerative capacity of the SI epithelium. For example, in pigs, PCNA^+^ cell quantities increased from birth during the suckling period and decreased as a result of weaning stress (Verdile et al., 2019). Furthermore, prolonged fasting and refeeding, which naturally occur in snakes, frogs, and fish, correlated with respective decreases and increases in PCNA^+^ intestinal epithelial cells (Helmstetter et al., 2009; Takahashi et al., 2014; Tamaoki et al., 2016). Chicken and other precocial birds exhibit dramatic changes in intestinal morphology and functionality during the peri-hatch period, in which their digestive tract undergoes a transition from embryonic, egg-based nutrition, to external nutrition (Uni et al., 1999, 2003b; Gilbert et al., 2007; Ding et al., 2011; Miska et al., 2014). Numerous studies have shown that the timing of first feeding, which may occur 24–72 h post-hatch in current poultry production practices, is critical for shaping the SI mucosa during the initial post-hatch period (Noy and Sklan, 1999; Bigot et al., 2003; Uni et al., 2003a).

We therefore sought to examine the quantity and spatial distribution of stem, progenitor, proliferating, and differentiated cells within the SI epithelium, in order to piece the puzzle of how specialized cell sub-types govern SI morphology and function, under the effects of EF, compared to 24 h DF, during several timepoints throughout the first 10d post-hatch. These timepoints will be further discussed.

### Hatch to d1

The first 24 h post-hatch is a period in which the quantities of egg-yolk nutrients diminish, and chicks become reliant on external feed for their development (Noy and Sklan, 1998; Yadgary et al., 2010). During this period, SI crypt development begins, and villi, that develop prior to hatch, expand and elongate (Uni et al., 2000, 2003b; Shyer et al., 2013).

Our work showed that lack of feed during this period resulted in stunted villus growth and decreased crypt cell counts. In contrast, feeding upon hatch maintained crypt cell counts and increased villi cell counts. This increase was not reflected in villi heights due to small villus cell dimensions during this 24 h period (Uni et al., 1998). The villus-crypt cell ratio was significantly higher at d1 than at Hatch, in both DF (un-fed) chicks and EF (fed) chicks. This indicates that during the first 24 h post-hatch, villi develop faster than crypts, but maintain a ≈5.5 ratio of villus cells per crypt cell, which was not affected by feeding. The timing of first feeding did not affect the ratios of stem, progenitor, and proliferating cells in the crypts. However, DF chicks exhibited decreased crypt differentiated cell ratios, indicating reduced functionality as a result of absence of feed during the initial 24 h. In the villi, ratios of proliferating cells were reduced in comparison to EF chicks and chicks at Hatch. Villi of EF chicks were composed of a higher number of FABP+ enterocytes and their goblet cell ratios were reduced, compared to DF chicks and chicks at Hatch. We therefore hypothesize that the halted development of villi of DF chicks may be attributed to reduced proliferation within the villi, and that feeding upon hatch maintained villus proliferation, resulting in a larger population of enterocytes, rather than goblet cells. Taken together, during the first 24 h post-hatch, external feed promotes the initial expansion of the digestive and absorptive surface area of the SI epithelium by supporting villus cell proliferation.

### d3

Between d1 and d3, the SI epithelium expanded in both groups, yet early feeding further promoted villus and crypt hyperplasia. EF chicks exhibited higher villi and crypt cell counts, compared to DF chicks. The villus-crypt cell ratio did not differ between groups, but decreased to ≈4. This indicates a shift at d3 toward a faster rate of crypt expansion, compared to villus elongation, and further emphasizes that this ratio is independent of the timing of first feeding. However, EF, compared to DF, shifted the ratios between crypt cell sub-types toward a decrease in crypt-base stem cell ratios, and increases in proliferating and differentiated cell ratios. Crypt cell sub-type ratios of DF chicks resembled those of chicks at d1, while those of EF chicks assumed a pattern more similar to later ages, which will be further discussed. In the villi, EF chicks exhibited higher counts and ratios of non-proliferating, FABP+ enterocytes, compared to DF chicks. Though crypt goblet cell counts and ratios were higher in EF chicks compared to DF chicks, their counts and ratios within the villi did not differ between EF chicks and DF chicks. Hence, EF promoted the differentiation of crypt cells into enterocytes, for further expansion of the digestive and absorptive area of the villi.

### d7 and d10

The SI mucosa of chicks was previously reported to reach morphological and functional maturity at days 7–10 (Uni et al., 1998, 1999). Accordingly, villus-crypt cell ratios stabilized to a ratio of ≈4.8 during these ages. The SI epithelium continuously expanded at d7 and d10, and crypt and villi cell counts remained higher in EF chicks, compared to DF chicks. This indicates that feeding upon Hatch, compared to 24 h-delayed feeding, elicits long-lasting hyperplasia of the SI epithelium.

At d7 and d10, crypts maintained a steady balance between Sox9+ stem cells and proliferating PCNA+ crypt cells, with lower stem cell ratios and higher proliferating cell ratios, in comparison to earlier timepoints. There were no differences in stem cell quantities and ratios between groups at these timepoints, in accordance with the similar expression patterns of Lgr5 mRNA in both groups at d10. Though several studies have found Lgr5+ crypt stem cells to co-express Sox9 (Gracz et al., 2010; Furuyama et al., 2011), a study by Roche et al. (2015) showed that varying levels of Lgr5 and Sox9 expression mark functionally different of crypt stem cells. Therefore, we conclude that by d10, the crypt stem cell population reaches a steady state in the chick SI epithelium and is no longer affected by the timing of first feeding.

In the villi at d7 and d10, the ratios of proliferating cells decreased in EF chicks, compared to DF chicks, and the quantities and ratios of mature enterocytes increased accordingly. This indicates that the increased digestive and absorptive surface of SI, as a result of EF, compared to DF, was maintained after 10 days. This was also evident by increased expression of enterocyte marker PepT1 in villi of EF chicks, compared to DF chicks, at d10. As for goblet cells, their ratios were higher in EF chicks compared to DF chicks at d7 and d10. This could be a result of the higher ratio of crypt goblet cells in EF chicks at d3, since crypt goblet cells give rise to the villus goblet cell population in a time-dependent manner (Paulus et al., 1993).

**Figure 7:**
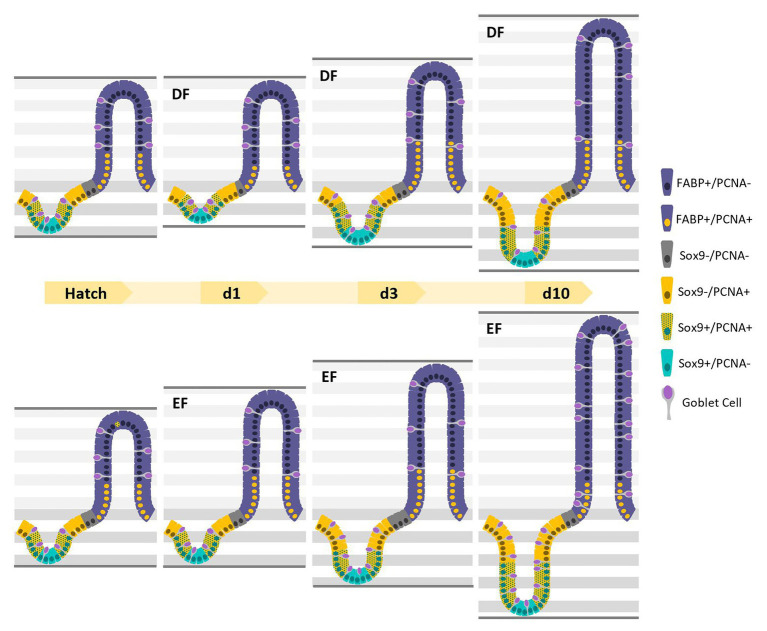
Timing of first feeding of chicks shapes the SI epithelium throughout development by altering the quantities and ratios of crypt and villus cell sub-types. Graphical representation of the major findings in this study. Villus cells were either positive for both FABP^+^ and PCNA^+^ or FABP^+^/PCNA^−^, and crypt cells were either positive for Sox9 and PCNA^−^, Sox9^+^/PCNA^+^, Sox9^−^/PCNA^+^, or Sox9^−^/PCNA^−^. Goblet cells were intermittently scattered throughout the crypt and villus epithelium. The effects of EF, compared to DF first feeding are represented by the total amount of villus and crypt cells and the quantities and ratios of each villus/crypt cell type, at d1, d3, and d10 (in which results resembled those of d7 in both groups).

Taken together, early feeding, compared to 24 h-delayed feeding, increased the amounts of villus enterocytes and goblet cells at d7 and d10, thus improving the SI functionality upon its maturation. These results demonstrate how a 24 h difference in the timing of first feeding elicits ongoing effects on the SI epithelium, which improve its functionality during the critical first 10 days post-hatch. Figure 7 summarizes the major findings in this study.

Our results provide further insights into the effects of the timing of first feeding on the cell sub-type quantities and distribution in SI epithelium, which generate and maintain SI morphology and functionality. Increased proliferation and differentiation shifted the SI epithelium into early maturation and enhanced functionality throughout the critical 10 days post-hatch period. These effects may be attributed to the contribution of specific nutrients to SI epithelial development, as indications for epigenetic programming of intestinal stem cell differentiation by dietary methionine have been reported (Obata et al., 2018). Moreover, the mechanical stimulation of the SI mucosa by luminal contents may also play a critical role in SI epithelial maturation. Studies of parenteral nutrition in humans and mice reported cellular atrophy and altered differentiation patterns in the SI epithelium in response to absence of mechanoluminal stimulation (Kovalenko and Basson, 2012; Wieck et al., 2017). In chicken, decreased villus heights due to fasting were recovered by enteral feeding, but not parenteral nutrition or non-nutritional luminal stimulation (Tarachai and Yamauchi, 2000). However, non-nutritional luminal stimulation improved weight gain in chicks susceptible to delayed feeding (Noy and Sklan, 1999). This demonstrates the contribution of luminal stimulation to the development and functionality of SI mucosa. Additionally, a growing body of evidence suggests that early feeding strategies can modulate the chick SI microbiota (Rubio, 2019). Since the gut microbiota affects SI structure and function (Dobrowolski et al., 2019), the timing of microbial introduction by external feed may also play a critical role in shaping the SI epithelium.

Although previous studies have demonstrated the effect of the timing of first feeding of chicks on growth, nutrient utilization, metabolic pathways, gut integrity, and immunity (Bigot et al., 2003; Bar Shira et al., 2005; de Jong et al., 2017; Hicks et al., 2019; Payne et al., 2019; Proszkowiec-weglarz et al., 2020), no study has shown the effects on crypt and villi epithelial cell sub-types, involved in cellular turnover and intestinal functionality. Our study is the first to show the effect of the timing of first feeding on the quantities and proportions of stem, progenitor, proliferating, and differentiated SI epithelial cells, through immunofluorescence and histochemical of specific cell sub-type markers.

The question whether the timing of first feeding elicits long-term effects on SI epithelial cell sub-type dynamics should be investigated in future studies. Mainly, whether early feeding promotes the differentiation of specific progenitor cells into particular epithelial cell sub-types associated with intestinal functionality, integrity, and immunity.

In conclusion, in chicks, early feeding influenced the dynamics between stem, progenitor, proliferating, and differentiated cells in the SI epithelium. Feeding upon hatch, as opposed to 24 h-delayed feeding (a routine procedure worldwide in the poultry production), elicited hyperplasia of progenitor and differentiated cells within the crypts and villi over the course of 10 days. This resulted in deeper crypts, higher villi, and a consequential expansion of the functional absorptive, digestive, and secretive surface area of the SI.

## Data Availability Statement

The raw data supporting the conclusions of this article will be made available by the authors, without undue reservation.

## Ethics Statement

The animal study was reviewed and approved by IACUC:AG-17-15355-2.

## Author Contributions

NR: PhD student, experimental design, execution, data anlysis, and manuscript writing. TM-Z and JD: experimental methodologies. ZU: supervision, funding, and manuscript review. All authors contributed to the article and approved the submitted version.

### Conflict of Interest

The authors declare that the research was conducted in the absence of any commercial or financial relationships that could be construed as a potential conflict of interest.

## Abbreviations

BSA: Bovine serum albumen
DAPI: 4',6-diamidino-2-phenylindole
DAB: 3,3'-Diaminobenzidine
IF: Immunofluorescence
ISH: *In situ* hybridization
Lgr5: Leucin-rich repeat-containing G-protein coupled receptor 5
PBS: Phosphate buffered saline
PCNA: Proliferating cell nuclear antigen
PepT1: Peptide transporter 1
PBST: Phosphate buffered saline, 0.1% Tween®
RT: Room temperature
SEM: standard error mean
SI: Small intestine
